# When should palliative care be introduced for people with progressive fibrotic interstitial lung disease? A meta-ethnography of the experiences of people with end-stage interstitial lung disease and their family carers

**DOI:** 10.1177/02692163221101753

**Published:** 2022-06-11

**Authors:** Evelyn Palmer, Emily Kavanagh, Shelina Visram, Anne-Marie Bourke, Ian Forrest, Catherine Exley

**Affiliations:** 1 Royal Victoria Infirmary, Newcastle upon Tyne, UK; 2 Marie Curie Hospice Newcastle, Newcastle upon Tyne, UK; 3 Population Health Sciences, Newcastle University, Newcastle upon Tyne, UK

**Keywords:** Interstitial lung disease, palliative care, meta-ethnography, qualitative research

## Abstract

**Background:**

Little is currently known about the perspectives of people with interstitial lung disease and their carers in relation to the timing of palliative care conversations.

**Aim:**

To establish patients’ and carers’ views on palliative care in interstitial lung disease and identify an optimum time to introduce the concept of palliative care.

**Design:**

Meta-ethnography of qualitative evidence. The review protocol was prospectively registered with PROSPERO (CRD42021243179).

**Data sources:**

Five electronic healthcare databases were searched (Medline, Embase, CINAHL, Scopus and Web of Science) from 1st January 1996 to 31st March 2022. Studies were included that used qualitative methodology and included patients’ or carers’ perspectives on living with end-stage disease or palliative care. Quality was assessed using the Critical Appraisal Skills Programme checklist.

**Results:**

About 1779 articles were identified by initial searches. Twelve met the inclusion criteria, providing evidence from 266 individuals across five countries. Three stages were identified in the illness journey of a person with interstitial lung disease: (1) Information seeking, (2) Grief and adjustment, (3) Fear of the future. Palliative care involvement was believed to be most appropriate in the latter two stages and should be prompted by changes in patients’ health such as respiratory infections, onset of new symptoms, hospital admission, decline in physical function and initiation of oxygen.

**Conclusions:**

Patients and carers prefer referral to palliative care services to be prompted by changes in health status. Future research should focus on supporting timely recognition of changes in patients’ health status and how to respond in a community setting.

**What is already known about the topic?**

- Despite the poor prognosis associated with progressive fibrotic lung disease, many people are not known to palliative care services or are referred late in the disease process.
- The experience of people with interstitial lung disease and their carers towards the end-of-life is poorly researched and it remains unclear when is the most appropriate time to involve palliative care services.

**What this paper adds?**

- Our study identified three interconnecting stages in the interstitial lung disease patient’s journey: information seeking, grief and acceptance, fear of the future.
- We suggest that palliative care involvement is most appropriate in the latter two stages and should be prompted by changes in patients’ health status.
- There is significant strain placed on the informal carers for people with interstitial lung disease.

**Implications for practice, theory or policy**

- Healthcare professionals must recognise changes in patients’ health status and initiate conversations which address palliative care needs and potential onwards palliative care referral.
- Future research should consider timely recognition of changes in patients’ health status and how to respond to these changes in a community setting.

## Introduction

Interstitial lung disease is a collective term for a group of conditions which cause scarring or inflammation in the lung interstitium.^1^ Progressive fibrosis is the end point of many types of interstitial lung disease and results in progressive respiratory failure. People with interstitial lung disease have significant symptoms with the most common being breathlessness, cough and fatigue.^2,3^ Idiopathic pulmonary fibrosis is the most common type of interstitial lung disease to cause progressive fibrosis and has an average life expectancy of 3–5 years, shorter than many cancers.^4–6^

Despite the high symptom burden and poor prognosis associated with progressive fibrotic interstitial lung disease, many patients are not known to specialist palliative care services or are referred late in the disease process.^7–10^ The experience of people with interstitial lung disease and their carers towards the end-of-life is poorly researched and it remains unclear when is the most appropriate time to involve palliative care services. It is important to optimise the timing of referral to specialist palliative care teams to help people with interstitial lung disease manage their symptoms and to facilitate discussion about end-of-life care. This review aimed to synthesise existing literature to establish patients’ and carers’ perspectives on the appropriate timing for introducing the concept of palliative care.

Previous systematic reviews have considered the timing of palliative care referrals in other non-malignant diseases.^11–13^ However, none of these reviews focussed on qualitative data to understand the patients’ perspective. We believe this is the first review to consider patient narratives to answer this important question from the viewpoint of people with interstitial lung disease and their families.

## Methods

### Study design

The review aimed to (i) identify qualitative studies involving people with interstitial lung disease and their carers and (ii) synthesise evidence about their perspectives of palliative care services. A study protocol was prospectively registered with PROSPERO (PROSPERO 2021 CRD42021243179). Synthesis was performed using an interpretative method of analysis to understand patients’ and carers’ perspectives about palliative care in interstitial lung disease. Meta-ethnography is a systematic method for synthesising qualitative evidence, which was first described by Noblit and Hare,^14^ and is widely used in healthcare research. The process involves seven steps which bring together findings from individual accounts to generate a ‘third order’ constructs^15^ (Table 1). As the included studies provided rich quotes to illustrate the themes developed in each study, meta-ethnography was selected as an appropriate method of synthesis. Reporting was performed according to the eMERGE meta-ethnography reporting guidance.^16^

**Table 1.** Seven stages of meta-ethnography.^a^

Phase of meta-ethnography	Description and application to the current study
Phase 1: Getting started	This involves identifying an intellectual interest that qualitative research might inform. Our study aimed to review patient and carer perspectives of palliative care in ILD, a research topic which was felt to be suited to qualitative literature review.
Phase 2: Deciding what is relevant to the initial interest	This comprises of identifying and selecting studies to include in the synthesis. Details of the search strategy and inclusion/exclusion criteria are included above.
Phase 3: Reading the studies	‘The repeated reading of the accounts and noting interpretative metaphors which requires extensive attention to the details in the accounts’.^a^
Phase 4: Determining how the studies are related	Determining the relationships between the studies by creating a list of the key metaphors, phrases, ideas and/or concepts used in each account and ‘juxtapose’ them.
Phase 5: Translating the studies into one another	The first level of synthesis; systematically comparing the meaning of metaphors, concepts or themes and their relations across study accounts.
Phase 6: Synthesising translations	The process of going beyond the findings or any individual study. The second level of synthesis where the translations are compared to identify common or overarching concepts and to develop new interpretations.
Phase 7: Expressing the synthesis	Communicating the synthesis to the audience in a suitable format.

a Noblit and Hare.^14^

### Search strategy

**Figure 1.**
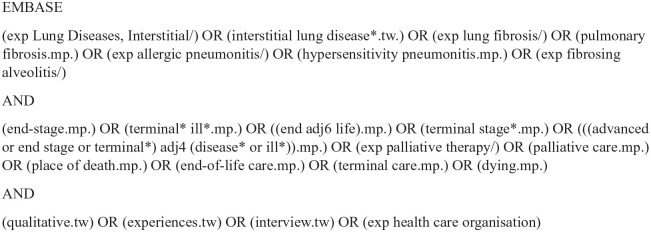
Example search strategy for the systematic review.

Five electronic databases were searched (Medline, Embase, CINAHL, Scopus and Web of Science) from 1st January 1996 to 31st March 2022. The search strategy (Figure 1 and Supplemental Material) was constructed with input from a medical librarian. Identification of qualitative literature through electronic database searching is challenging,^17,18^ therefore we supplemented our database search by handsearching the reference lists of included studies. The date range was limited to articles published after 1996 as the management of patients with interstitial lung disease and palliative care services have substantially changed in the last 25 years and the aim was to focus on contemporary literature. Inclusion and exclusion criteria are presented in Table 2. No exclusions were made based on country of publication, although only studies published in English were included.

**Table 2.** Systematic review inclusion and exclusion criteria.

Inclusion criteria	Exclusion criteria
Original research using and reporting qualitative methodologies	Studies which did not report qualitative methods for data collection and analysis. Mixed methods studies were included if they reported primary qualitative data.
Studies published in English	Studies published in any other language
Studies that included the perspectives of adult patients (aged >18 years) with a diagnosis of interstitial lung disease and/or their carers about palliative care or living with end-stage disease.	Studies which did not focus on patients with interstitial lung disease and/or their carers Studies which did not mention end-stage disease or palliative care Conference abstracts and dissertations/theses

### Data collection

The results of the database searches were exported into EndNoteX9 where they were merged, and all duplicate studies removed. Two researchers (EP and EK) independently screened the titles and abstracts of the retrieved studies for eligibility. Full texts were then obtained and evaluated for studies which were thought to meet the review criteria. These were reviewed by both researchers to determine study inclusion and any disagreements were resolved by consensus.

### Quality assessment and risk of bias

Two researchers (EP and EK) independently assessed the methodological quality of the included studies using the Critical Appraisal Skills Programme (CASP) checklist for qualitative studies.^19^ Based on these tools an assessment of the methodological quality was made as ‘Excellent’, ‘Good’, ‘Fair’ or ‘Poor’. No studies were excluded based on the quality assessment; however, it was used as a variable in the subsequent analysis and studies with ‘poor’ methodology were given less weighting in the analysis.

### Data analysis and synthesis

Data analysis was performed by EP following the seven stage meta-ethnography approach described by Noblit and Hare^14^ (Table 1). Reading and re-reading the included studies was undertaken and a predefined data extraction matrix was completed which contained the following information: general study characteristics, methods, participants, results (main themes identified in each study and concepts from our scoping review). Completion of the data matrix allowed visual representation of how concepts and ideas were related between studies. The first (quotes from the studies) and second (themes developed by primary authors) order constructs were analysed through constant comparison,^20,21^ identifying that the studies had reciprocal results (Supplemental Material). A reciprocal translation synthesis was performed. Key components and experiences were translated between the studies, ensuring that no relevant or contradictory findings were ignored. The interpretive process and development of the conceptual analysis were iterative and reviewed the data extraction matrix and primary studies to ensure findings were consistent with the original data.^22^ The second order constructs connected and contributed to the development of a new level of interpretation (lines of argument) based on the patient and carer experience of interstitial lung disease from time of diagnosis to their death.

The lead author (EP) assumes a critical realism paradigm position which will have influenced the interpretation of findings during data analysis.

## Results

### Study characteristics

**Figure 2.**
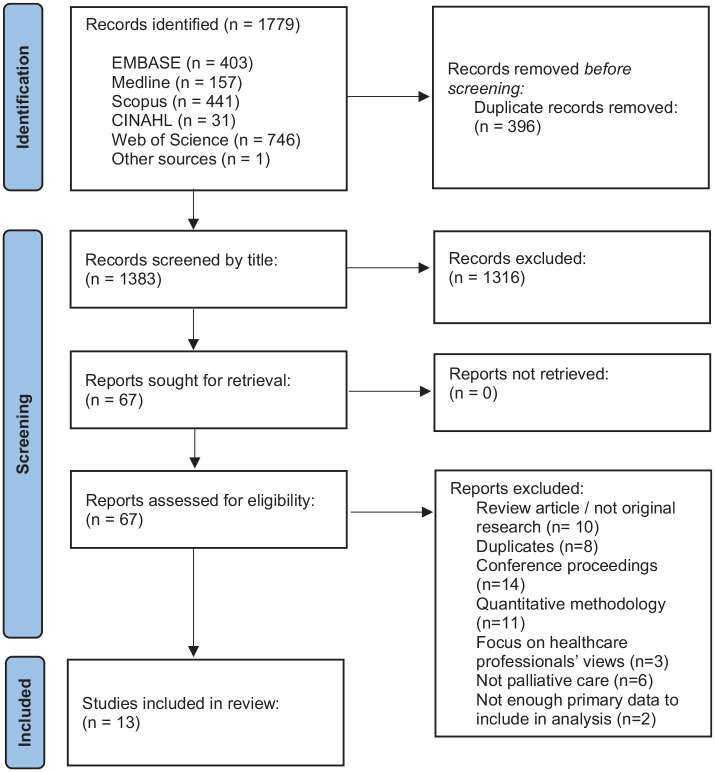
PRISMA 2020 flow diagram for new systematic reviews which included searches of databases and registers only. Page et al.^29^ For more information, visit: http://www.prisma-statement.org/. ILD: interstitial lung disease.

The search identified 1779 publications, resulting in 67 full text articles reviewed. Of these, 12 articles, presenting data from 11 studies, were included in the final analysis (Figure 2). The included studies involved 118 people with interstitial lung disease and 118 informal carers of people with interstitial lung disease (Table 3). Although it was not one of the specific inclusion criteria, the included studies also involved 30 health care professionals. The studies were undertaken in the UK (*n *= 3), Europe (*n* = 2), USA (*n* = 2), Australia (*n* = 1) and Canada (*n* = 3). Nine studies generated data through qualitative interviews and two from focus groups. Seven of the studies only included participants who had idiopathic pulmonary fibrosis or their carers, whereas the other four studies also included other types of interstitial lung disease. Two of the UK articles presented data from the same interview subjects^23,24^ two papers were written by the same research group in Denmark^25,26^ and a further three by a research group in Canada.^27,28^ All the papers were published between 2013 and 2021 reflecting the increased interest in qualitative research in this field in recent years.

**Table 3.** Characteristics of included studies and quality assessment score.

Study number	Author	Aim of study	Population (country)	Study design	Quality assessment	Key findings of the study
1	Bajwah et al.^23^	Explore the specialist palliative care needs of people living with end-stage progressive fibrotic ILD.	ILD patients *n* = 8 Carers *n* = 4 Healthcare professionals *n* = 6 (UK)	Qualitative interviews	Excellent	• Profound physical and psychological symptom burden – the main symptoms were shortness of breath, cough and insomnia • Illness progressively prevented patients going about their everyday lives, many patients felt isolated and lonely with their identified consumed by their illness • Increasing reliance on others and change in their relationships • Resignation among healthcare professionals that patients would inevitably suffer poor symptom control • Informal carers provide invaluable support to patients and often enable them to stay in their preferred place of care • Psychological needs of patients and carers are not currently being met
2	Bajwah et al.^24^	Explore understanding of disease, preferences re EOLC and views on communication + coordination of care for patients with ILD	ILD patients *n* = 8 Carers *n* = 4 Healthcare professionals *n* = 6 (UK)	Qualitative interviews	Good	• Patients and carers had a limited understanding of their disease, its poor prognosis or how the disease would manifest in the end stages • Unmet information needs about end-of-life care • None of the patients or carers had formulated any end-of-life plans • Identified problems with communication between healthcare professionals and organisation of care services
3	Belkin et al.^40^	Capture informal caregivers’ perspectives on how they are affected by having a loved one with IPF.	Carers *n* = 14 (UK)	Focus groups	Excellent	• Informal carers face a struggle between performing their duties as a caregiver and maintaining their own identities and independence • IPF was perceived as thoroughly dominating patients’ lives • Patients feared disease progression and using denial as a coping mechanism • Oxygen was viewed with trepidation, as a marker of disease progression and major life change
4	Duck et al.^35^	Understand the perceptions, needs and experiences of patients with IPF	IPF patients *n* = 17 Carers *n* = 6 (UK)	Qualitative interviews	Good	• Many patients struggled to get a diagnosis which had a negative effect on their wellbeing • Participants experienced loss of independence, changing roles in relationships and loss of spontaneity • Patients struggled with progressive symptoms as IPF affected every aspect of their lives – experiencing functional limitation, rapid disease progression with limited support and few positive treatment options. • Despite oxygen being a symbol of deteriorating disease, most patients described that it enabled them to do the things they wanted. • Patients who were waiting for a lung transplant or in a clinical trial were more hopeful than other patients.
5	Egerod et al.^26^	Investigate the experience of relatives during the final stages of illness and first year after patient’s death.	Bereaved carers *n* = 20 (Denmark)	Qualitative interviews	Good	• The timing, location and process of death were pivotal for the carers’ experience • Sudden death could leave a carer with unanswered questions • Not all patients had preferences for location of end-of-life care, but it was important that the patient died at the location of choice or at a familiar location • The ideal process of dying was a comfortable patient with their family present • The process of grief started at the time of diagnosis, when carers began to prepare for their impending loss • Many carers experienced worsening health after their spouses’ death
6	Holland et al.^38^	Explore the perspectives of patients and ILD clinicians regarding the educational content of pulmonary rehabilitation for ILD	ILD patients *n* = 18 Healthcare professionals *n* = 14 (Australia)	Qualitative interviews	Good	• Patients wanted clinicians to be honest about prognosis and provide information about what they should expect over the natural course of the disease • Most patients were interested in hearing information about planning for the end of life during PR • Clinicians identified management of cough and oxygen therapy as key areas for ILD education in PR • There was no consensus amongst clinicians about whether PR was an appropriate setting to discuss end of life planning with some strongly advocating that these discussions should only be held in private
7	Kalluri et al.^39^	Explore perspectives of IPF patients, family caregivers and healthcare professionals on advance care planning related experiences.	IPF patients *n* = 5 Carers *n* = 5 Healthcare professionals *n* = 10 (Canada)	Qualitative interviews	Good	• Patients, carers and healthcare professionals perceived there was ‘insufficient information’ about ACP and that ‘conversations occur late’. • Recommendations identified to improve ACP included: have earlier conversations, provide information, have open conversations and plan for EOLC. • Patients and carers felt ACP conversations should be with a respiratory specialist. • HCPs should discuss available EOL options, including home and hospice.
8	Kalluri et al.^27^	Explore perceptions of symptoms and symptom management strategies among people with IPF and their carers who received self-management education and action plans.	IPF patients *n* = 13 Carers *n* = 8 (Canada)	Qualitative interviews	Excellent	• Participants used a range of strategies; physical, cognitive, breathing and oxygenation and safety and environmental • Most participants were confident in managing their symptoms and reported fewer symptoms • Both patients and carers were able to shift perspectives to focus on living well with IPF • The majority of participants had engaged with anticipatory planning with medical and financial plans in place for the end of life
9	Lindell et al.^36^	Explore the perceptions of palliative care needs in patients with IPF and their caregivers	IPF patients *n* = 5 Carers *n* = 5 Bereaved carers *n* = 3 (USA)	Focus groups	Good	• Frustration with diagnostic process and education received • Overwhelming symptom burden • Patients and carers expressed reluctance to engage in advance care planning • Comfort in the care provided by speciality centre because of the resources available (e.g. support group, involvement in research, potential to pursue lung transplant)
10	Overgaard et al.^25^	Increase knowledge of life with IPF for patients and family caregivers.	IPF patients *n* = 25 Carers *n* = 24 (Denmark)	Qualitative interviews	Fair	• IPF patients need more information at the time of diagnosis, but information should be paced as the disease progresses • Carers need to be supported in their role in caring for their spouses • Carers were perpetually vigilant and observed patients continuously, fearing that exacerbations of symptoms would be fatal • Patients and carers avoided the issue of decline, and carers gradually and tacitly spouses took over chores • Patients coped with deterioration by trying to maintain normal life for as long as possible • Oxygen was seen as a negative milestone in the illness trajectory
11	Pooler et al.^28^	Explore bereaved caregivers’ experiences + perceptions of an early integrated palliative approach implemented by an multidisciplinary ILD clinic	Bereaved carers *n* = 8 (Canada)	Qualitative interviews	Fair	• Collaboration and close communication between carers, clinicians and home care enabled effective symptom management and out of hospital deaths • Patients and carers struggle to obtain a diagnosis and were not informed of the poor prognosis until they were seen in a specialist clinic • Early and ongoing advance care planning conversations enabled patients to express their goals and wishes • Management of symptoms enabled patients and carers to have a better quality of life and experience meaningful activities together • Carers experienced strain and responsibility when caring for their spouses at home, due to physical work, insufficient healthcare resources and limited knowledge of community support. • Carers described a sense of achievement and gratification from keeping their spouses out of hospital in accordance with their wishes
12	Sampson et al.^37^	Address uncertainties relating to the care needs of patients and carers at different stages in the IPF disease trajectory	IPF patients *n* = 27 Carers *n* = 21 (UK)	Qualitative interviews	Good	• Patients with IPF have a clear understanding of their prognosis, but limited understanding of how their disease will progress and how it will be managed. • Patients need to receive information at an appropriate pace triggered by changes they perceived in health status – this did not always coincide with ILD clinic appointments • The role of carers needs to be acknowledged in outpatient consultations and enable carers to play an active role throughout the patient pathway • Early MDT support should be triggered by changes in health status – for example, initiation of oxygen, worsening symptoms, changes in physical or social functioning • Recognition that patients and carers differ in their information needs and that these needs change over time

ILD: interstitial lung disease; EOL(C): end of life (care); SPC: specialist palliative care; HRQoL: health-related quality of life; MDT: multidisciplinary team; DNAR: resuscitation order; ACP: advance care planning; PPD: preferred place of death; PR: pulmonary rehabilitation.

### Quality appraisal

There is controversy about whether quality appraisal should be performed in meta-ethnography.^30–32^ It was decided to undertake quality appraisal to examine the limitations of each article in detail prior to synthesis. The quality of each article based on the qualitative CASP assessment tool are summarised in Table 4. Three studies were assessed as excellent, seven as good and two were fair quality. No studies were excluded based on the CASP score, as there is no evidence that this improves the quality of the review.^30,33^

**Table 4.** Summary of quality appraisal using CASP qualitative assessment tool.

Study	Clear statement of aims	Qualitative methodology appropriate	Research design appropriate for aims	Recruitment strategy appropriate for aims	Data collection appropriate for aims	Relationship between researcher and participants considered	Ethical issues considered	Data analysis rigorous	Clear statement of findings	Overall rating
Study 1: Bajwah et al.^23^	Yes	Yes	Yes	Yes	Yes	Yes	Yes	Yes	Yes	Excellent
Study 2: Bajwah et al.^24^	Yes	Yes	Yes	Yes	Yes	Can’t tell	Yes	Yes	Yes	Good
Study 3: Belkin et al.^40^	Yes	Yes	Yes	Yes	Yes	Yes	Yes	Yes	Yes	Excellent
Study 4: Duck et al.^35^	Yes	Yes	Yes	Yes	Yes	Can’t tell	Yes	Yes	Yes	Good
Study 5: Egerod et al.^26^	Yes	Yes	Yes	Yes	Yes	Can’t tell	Yes	Yes	Yes	Good
Study 6: Holland et al.^38^	Yes	Yes	Yes	Yes	Yes	Can’t tell	Yes	Yes	Yes	Good
Study 7: Kalluri et al.^39^	Yes	Yes	Yes	Can’t tell	Yes	Yes	Yes	Yes	Yes	Good
Study 8: Kalluri et al.^27^	Yes	Yes	Yes	Yes	Yes	Yes	Yes	Yes	Yes	Excellent
Study 9: Lindell et al.^36^	Yes	Yes	Yes	Yes	Yes	Yes	Can’t tell	Yes	Yes	Good
Study 10: Overgaard et al.^25^	Yes	Yes	Yes	Can’t tell	Yes	Can’t tell	Yes	Yes	Yes	Fair
Study 11: Pooler et al.^28^	Yes	Yes	Yes	Yes	Yes	Can’t tell	Yes	Can’t tell	Yes	Fair
Study 12: Sampson et al.^37^	Yes	Yes	Yes	Yes	Yes	Can’t tell	Yes	Yes	Yes	Good

Overall quality assessment	
Excellent	All responses ‘Yes’
Good	One ‘Can’t tell’ response
Fair	One ‘No’ response or two ‘Can’t tell’ responses
Poor	More than one ‘No’ response or more than two ‘Can’t tell’ responses

### Synthesising translations: Lines of argument

**Figure 3.**
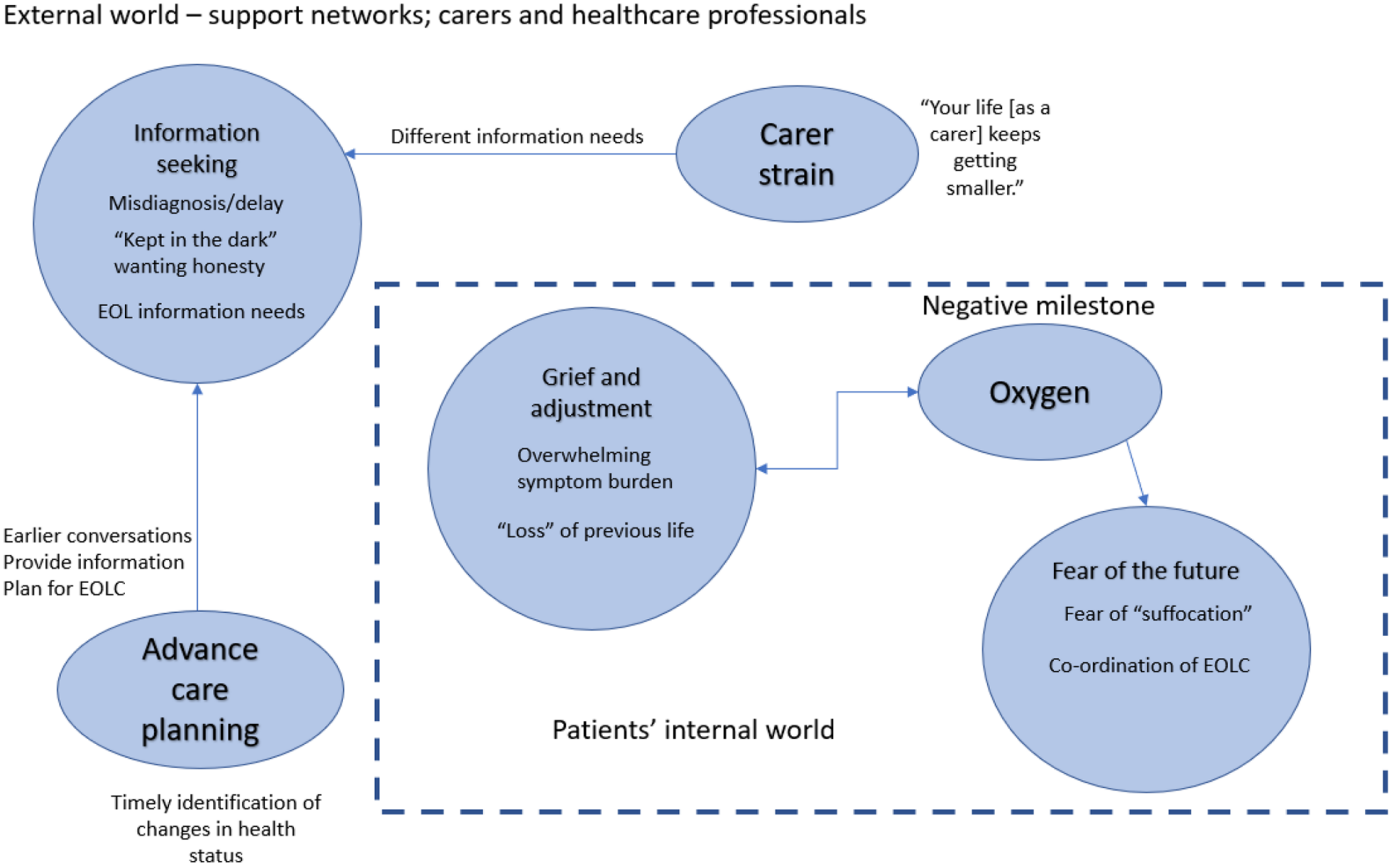
Conceptual model of lines of argument synthesis.

**Figure 4.**
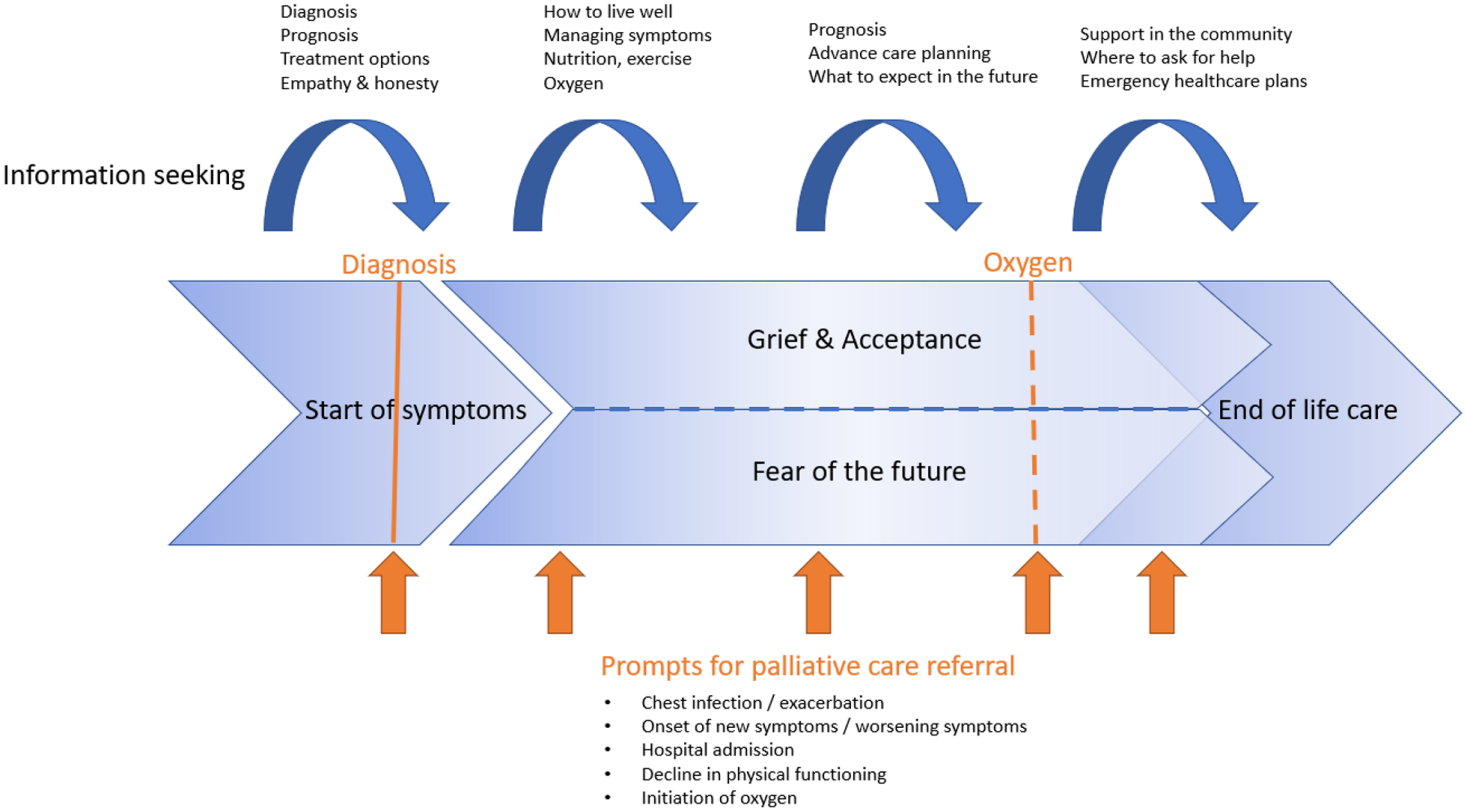
The patient journey and prompts for considering palliative care referral.

The studies all related to one another through contribution to a lines of argument synthesis (Figure 3) encompassing three multifaceted overarching key concepts: (1) information seeking, (2) grief and acceptance, (3) fear of the future. In the following sections, these key concepts and their associated sub-concepts are discussed. Figure 4 depicts how these key concepts interact through the patient and carer illness journey.

### Information seeking

Misdiagnosis and delay in referral to speciality centres were common themes from the included studies. Many people were unsuccessfully treated for alternative lung conditions before the diagnosis of interstitial lung disease was eventually established.^25,35,36^

*“It started about 5 years ago, but I didn’t realise then that something was terribly wrong. It just came gradually. I was diagnosed with something “fibrotic” and was told that I probably had untreated asthma”. (patient study 10)*

There was frustration at the lengthy and complicated diagnostic process.^37^ Following diagnosis, some people were not referred to speciality centres until late in the disease as they were not deemed ‘sick enough’. This delay had a significant impact on patient care; one carer reported that his wife died before being seen in the specialist clinic and another person missed out on inclusion in a clinical trial due to delayed referral.^35,36^ Delayed diagnosis and referral contributed to the feelings of frustration and helplessness experienced by patients who were seeking information about their lung condition.

People with interstitial lung disease wanted more information about their lung disease and felt this was particularly important around the time of diagnosis.^36,37^ Staff at the specialist clinic were seen as a trustworthy source of information^36–38^ and talking to someone who knew about their condition gave patients confidence in the speciality centre.^36^ However, patients were frustrated with the lack of knowledge about interstitial lung disease outside of speciality centres which contributed to the burden of uncertainty about future management.^36,37,39^

*“It was so hard to find information and any doctors or nurses we did talk to had never had a patient with IPF before [. . .] they were comparing it to COPD or other diseases that it really can’t be compared to. There was no information and it felt like we were completely on our own. . .From what I found online, it was scary; but I figured if that was the case, then her doctor would tell us”. (carer, study 7)*

Patients preferred to receive written information, and although many did research their condition on the internet this was felt to be confusing and sometimes portraying ‘worst case scenarios’.^37,38^ Although the speciality clinics were seen as a reliable source of information, many patients were dissatisfied with the level of information they received about their diagnosis and how to manage their disease.

Information needs changed throughout the course of the disease trajectory. When symptoms started and at the time of diagnosis, patients wanted information on their lung disease, prognosis and potential treatment options.^35,38^ ‘*I think at diagnosis, obviously, you need to be told that this is not curable and it’s progressive and it will end your life’ (patient with IPF, study 7)*. As time passed and the disease progressed, patients expressed a need for information about oxygen therapy, nutrition, exercise and management of cough and breathlessness symptoms.^37,38^ Pulmonary rehabilitation was viewed as a positive intervention, a way to learn more about their disease and to focus on how they could maintain their health.^37,38^ *‘I want to know what I should do to prevent it from getting worse and what is the good exercise for this situation’. (patient with IPF, study 6)*. People with interstitial lung disease want more education about their disease and to focus on lifestyle changes that can help them manage their disease and symptoms. A Canadian study which reviewed the effect of self-management education found that patients were more confident managing their symptoms and able to shift their perspectives to focus on living well with their disease.^27^

People with interstitial lung disease and their carers expressed a strong desire for more specific information about disease management towards the end-of-life.^24,37,38^ Holland et al.^38^ discussed the inclusion of information about end-of-life care in pulmonary rehabilitation, which was widely acceptable to patients; however, some healthcare professionals had concerns about discussing this topic in a group environment. Two Canadian studies that interviewed patients and bereaved carers described the benefits of advance care planning which helped patients and their carers live their life fully whilst preparing for death.^28,39^ Conversely, a US focus group study which included both patients and carers reported confusion about the goals of palliative care and that the need for care planning was recognised but avoided as it was perceived as a loss of hope.^36^ Pooler et al.^28^ counteract this view and reported that carers felt supported by advance care planning conversations, which were presented as strategies to help patients live life well within the limitations of their disease.

Desynchrony in the information needs of patients and carers was identified throughout the patient journey but was most apparent in relation to information about end-of-life planning.^37^ Carers often wanted practical advice about how to manage symptoms and what to expect towards the end-of-life.

*“it’s hard to anticipate what help I need if I don’t know what’s coming. . . I never know how doctors feel about a spouse sitting there and suddenly taking over the conversation, so I generally don’t ask questions. I answer questions if I’m asked. . .but I think that I’m there just as a support system to [partner], rather than having my own questions answered”. (carer, study 12)*

Whereas patients did not always want to discuss things in the same level of detail. Patients who lived alone were more direct in their need for information about the future course of the disease and available sources of support.^37^ These conflicting views about end-of-life information needs for people with interstitial lung disease demonstrate the difficulty experienced by healthcare professionals when initiating advance care planning conversations.

There was no agreement from the included studies about the optimal time for involving palliative care in patient management. Kalluri et al.^27^ described the benefits of an early integrated palliative care approach combined with patient self-management strategies. Clinicians were trusted to decide on when was the right time to initiate discussions and how much information should be given and at what time.^24^ However, some people with interstitial lung disease preferred not to know everything at once and receive prognostic information gradually and gently,^25^ whereas other people reported a need for honesty from the start.^38,39^ Patients wanted to receive information at an appropriate pace, triggered by changes in their perceived health status, rather than dictated by the timing of clinic appointments.^37^ Most patients were happy to receive information about interstitial lung disease from any suitably qualified healthcare professional, however, a small minority preferred only to speak to a specialist physician.^38,39^ ‘*A general practitioner is not up to it, it’s not their speciality’*. *(patient with IPF, study 7)* Healthcare professionals recognised the importance of empowering patients and their carers with information about their condition but also appreciated that these were difficult conversations and needed to be approached sensitively.^24^

*“I think sometimes the delivery is wrong (. . .) which could be a problem. Um what you don’t want to do is completely say ‘you’re going to die’ what you want to do is give them some hope”. (ILD nurse specialist, study 2)*

It was important to patients that difficult and potentially upsetting conversations should take place in person with their family present and with a trusted member of the clinical team.^25^ Healthcare professionals must appreciate the sensitive nature of conversations about prognosis, stopping treatment and introducing palliative care and ensure that people important to the patient are present and to allow adequate time for these conversations.

### Grief and adjustment

People with interstitial lung disease need time to understand their diagnosis, the impact it will have on their life, and to grieve for the ‘loss’ of their previous life. Some felt cheated out of their retirement plans,^23^ and others mourned the activities which they enjoyed and in which they could no longer participate.^23,35^

*“I can’t go anywhere [. . .] I don’t [really] have a life I’m sitting indoors everyday. . .I used to be meet friends and have coffee and it [would] give you a bit of life back”. (female patient in her 70s, study 1)*

Changes in roles and responsibilities around the house were particularly significant to patients. Many found it difficult to be looked after by their partners and had to supervise activities such as DIY or mowing the lawn rather than these tasks being their responsibility.^25,35^

*“I’ve always been active, garden, DIY, you know I’d do anything around the house. I mean there was a little job there yesterday and I had to get Christine to (do it). . .I sort of direct operations now, but physically do it, No”. (patient, study 4)*

These significant changes led to people with interstitial lung disease feeling isolated, lonely and lacking purpose as their lives and identities had been consumed by their illness.^23^ However, a study which focussed on the introduction of self-management strategies depicted a very different outlook, with patients describing feeling positive about their symptom control and concentrating on meaningful activities.^27^ This study recruited patients from a specialist clinic with early integrated palliative care and an extensive patient education programme, a situation which may explain the incongruence of these results.

Many studies which included carer participants highlighted the significant strain on family relationships, which stemmed from the diminished functional ability associated with interstitial lung disease.^23,25,26,40^ *‘He can’t do a thing, we don’t talk about it. I do what has to be done’. (carer study 10)* Family carers expressed frustration, both at the difficulty of having to ‘do everything’ and look after their spouse with little respite, but also at the life that they had lost.^25,40^ *‘Your life [as the informal carer] keeps getting smaller, which is really difficult’. (carer, study 3)* Carers also reported that their partners persistently talked about their health and took their frustration and anger out on their spouses which led to further strain on relationships.^23,40^ Carers expressed feeling helpless at their inability to relieve their loved ones suffering.^40^ The process of accepting their diagnosis and adjusting to the limitations that symptoms placed on their lives was different for everyone with interstitial lung disease. Informal carers also carry the burden of these limitations and may require additional support from healthcare professionals.

### Fear of the future

The studies identified in this review, showed that people with interstitial lung disease were fearful of disease progression, continued loss of independence and dying. Many did not feel that their fears about the future were adequately addressed in clinic appointments.^38^

*“Being honest and telling me what’s going to happen. It’s very important. It’s one of my biggest fears [. . .] I do feel much better just knowing, I was afraid of the end. Of how violent it could be”. (patient with IPF, study 7)*

The relevance of disease-focussed assessments in clinic, such as lung function tests, were difficult for people to interpret and relate to their symptoms,^37^ and instead disease progression was measured by their loss of functional ability over time.^35^ Oxygen was viewed with trepidation as a negative milestone which indicated disease progression.^25,40^

*“We are not dealing with oxygen yet, and I hope we can put that off a long time, but in reality, we know it probably will happen at some point.. . .it is terrifying to me and to him”. (carer, study 3)*

Patients and carers held differing views about oxygen; with carers viewing oxygen as enabling patients to do more activities and patients viewing oxygen as a ‘failure’.^37,40^ This divergence led to tensions and conflict when carers attempted to encourage the use oxygen to facilitate activity.^36^ Conversely, two studies reported the views of patients who were accepting of oxygen and the benefits of using this to help with their daily lives^27,35^ These differing perspectives highlight the importance of open, honest discussion with patients to address their specific fears and not assume that clinicians understand the complexity of these concerns.

People with interstitial lung disease were accepting of the fatality of their diagnosis but feared the dying process. Patients feared ‘suffocation’ and family members often did not know what to expect at the end-of-life.^26^ *‘I knew he wasn’t afraid of dying. He said so, but he was afraid of the process, and worried that he would suffocate’. (carer, study 5)* Many people with interstitial lung disease wanted to die at home, and in some of the studies this was made possible by the support of their carers and clinical team.^26,28^ One study which included early palliative care involvement reported that the majority of participants had medical and financial plans in place for the end-of-life.^27^ However, other studies reported reluctance to engage with advance care planning and in some cases unrealistic expectations about what would happen in the future.^24,36^ Egerod et al.^26^ interviewed bereaved carers of people with interstitial lung disease and concluded that the location, timing and the process of death had an impact of the carers’ grief reaction. Carers whose family member had died in hospital appeared to be less prepared for their death and were shocked by how quickly they died.^26^ The burden on informal carers providing end-of-life care is significant and can affect their memories of those final weeks and days.^26,28^

*“I found [dying at home] difficult, because he was very ill and it required a lot of work. I didn’t always have the stamina or knowledge of how to manage, so sometimes we barked at each other. When I look back, I should have had help. . ..so we could have enjoyed each other”. (carer, study 5)*

Most carers felt gratification from enabling their loved ones to die at home. Breakdown in the communication between healthcare providers and patients and their families increased the stress of caring for a dying relative.^24,26^ *‘They promised that he would go to his usual hospital, but suddenly one morning the nurses said that he wouldn’t go there. I actually think he got angry and then he couldn’t breathe’. (carer, study 5)* Support from healthcare professionals is crucial in reducing the burden on informal carers and allowing them to spend quality time with their loved ones.

## Discussion

This systematic review aimed to establish patients’ and carers’ perspectives on the timing of involvement with palliative care services for people with interstitial lung disease. We identified three key stages which occur in the illness journey for patient with interstitial lung disease; (1) Information seeking, (2) Grief and adjustment, (3) Fear of the future. In our discussion we highlight several recommendations to improve the experience of palliative care for people with interstitial lung disease and their carers and how the timing of palliative care interventions can be tailored to these stages in the illness journey.

## What is already known

Our review found that people with interstitial lung disease were often subjected to a lengthy and complicated diagnostic pathway before their diagnosis was confirmed. Diagnostic delay has been attributed to overlap with other chronic lung conditions, treatment for alternative diagnoses, underreporting of interstitial lung disease features on diagnostic investigations and poor understanding of the condition in primary care.^41–43^ Delay in referral to speciality centres has been associated with increased mortality in interstitial lung disease.^44,45^ Our findings are corroborated by the results of a European survey of patients with idiopathic pulmonary fibrosis who also described a protracted route to obtaining a diagnosis.^46^ In this survey, patients viewed their initial diagnosis of asthma, COPD or pneumonia, not as a working hypothesis, but as an error or misdiagnosis. Understandably, diagnostic delay and perceived misdiagnosis had an impact on how people with interstitial lung disease viewed the quality of their care.

This review identified that many patients were dissatisfied with the information they received about their condition, particularly around the time of diagnosis.^47–49^ Our synthesis suggests that alongside in-person individualised conversations in clinic, written information is preferred by people with interstitial lung disease who had difficulty accessing internet resources and found the content confusing. Patients and carers want information to be available in multiple modalities which they can use as reference to help them feel in control of the management of their disease.^49^ People with interstitial lung disease want clinicians to provide information which promotes the importance of active self-care including exercise, nutrition and management of chest infections.^48^ Written information provided at the time of diagnosis should include signposting to further information about palliative care, which may empower patients to discuss this further at subsequent appointments. This concept of ‘layered information sources’ was also suggested by an European focus group study which centred on communication difficulties reported by people with idiopathic pulmonary fbibrosis.^50^ This would introduce the concept of palliative care, whilst maintaining a positive outlook at the initial consultation, allowing patients time to understand, research and accept their new diagnosis and the impact this would have on their future health. Although this approach allows for a small amount of generic palliative care information to be made available to everyone at the time of diagnosis, health care professionals should discuss with patients if they felt that exploring the palliative care information was specifically relevant at that point in their disease trajectory.

Most people with interstitial lung disease understood the gravity of their diagnosis, particularly those who had been diagnosed with idiopathic pulmonary fibrosis. They experienced grief for the ‘loss’ of their previous life, loss of spontaneity and increasing dependence on family caregivers. ‘Loss of self’ is a well described phenomenon in chronic illness where patients observe their former self-images disappearing without the simultaneous development of an equally valued new versions.^51^ The adjustments to household roles were seen by many people with interstitial lung disease as symbolic of the physical constraints of their illness. Changes in spousal relationships and household roles are common in chronic disease where the spouse takes on an informal carer responsibility.^52,53^

Our study identified oxygen as a ‘negative milestone’ which patients viewed with trepidation as it indicated a significant deterioration in their lung disease. Oxygen has been identified as a marker of disease progression in interstitial lung disease^10^ and the point where the disease becomes highly visible to others.^46^ Uncertainty about the purpose and proposed benefits of oxygen, physical difficulty using devices, and embarrassment about using oxygen in public have been reported by patients with chronic lung disease.^54–56^ Education about oxygen, how it should be used, and the benefits of this treatment should be discussed with all patients. This may be better received prior to the point at which oxygen prescription is required as patients can take some time to come to terms with reaching this negative milestone. Honest and empathic discussion at this time would acknowledge patients’ fears about oxygen and could introduce palliative care as another way to help manage symptoms and improve quality of life.

Many people with interstitial lung disease feared the future; this was evident form the time of diagnosis for some people and others only as their disease progressed. Fear of suffocation was a common theme from the included studies which has been reported in other chronic lung conditions^57,58^ and can lead to worsening respiratory discomfort.^59^ Healthcare professionals need to find a way to identify and address these fears about the future. Conversations about dying should go beyond the focus on resuscitation and address the wider concerns of patients and their families; including fears about dying, understanding their prognosis, achieving their end-of-life goals and attending to their physical needs.^60^ There is evidence that advance care planning conversations positively impact the quality of end-of-life care,^61,62^ however, these conversations are surprisingly uncommon for patients with advanced respiratory disease.^63^ The role of respiratory physicians has evolved and should embrace the challenges of addressing patients’ fears about the future and initiating advance care planning conversations.^64^ Engagement in advance care planning can relieve some of the anxiety and uncertainty experienced by people with interstitial lung disease and alleviate strain felt by informal caregivers.

## Strengths and limitations of the literature

The search strategy is a strength of this analysis; the searches were conducted across five research databases with review of the reference lists of included studies to identify additional relevant studies. The included studies originated from the UK, Denmark, USA, Canada and Australia. This increases the international relevance and transferability of the findings, particularly as the studies reported reciprocal results. Overall, the methodological assessment of the included studies was positive with none of the studies being rated as ‘poor’. This adds to the certainty and robustness of this synthesis as it included high quality primary studies which reported congruent results.

The number or participants in the studies ranged from 8 to 49. The study which included only eight participants was a review of bereaved carers’ experiences of involvement with an early integrated palliative approach implemented at a multidisciplinary clinic. Therefore, the results of this study are less generalisable to the overall interstitial lung disease population who would not have access to this type of service. All studies recruited patients from tertiary centres in high income countries and therefore the results may not reflect the patient and carer experience in lower income countries, smaller hospitals or those who live further away from specialist centres.

## Strengths and limitations of the meta-ethnography

The interpretive nature of meta-ethnography mean that the results of this review are open to criticism. Other researchers with different philosophical and professional experiences could have obtained a different synthesis. An important limitation is the reliance on the data presented in the included studies, which may not fully reflect the original data. However, a strength of meta-ethnography is the generation of ‘third order’ constructs and new evidence to answer questions about the experiences of people with end-stage interstitial lung disease and their carers.

## What our study adds

Our study aimed to synthesise qualitative data about the experiences of people with interstitial lung disease and their carers to recommend the appropriate timing in the patient journey to introduce palliative care. Previous research has suggested that early involvement of specialist palliative care for people with interstitial lung disease is desirable.^65,66^ Our study identified that although there were some patients who felt palliative care discussions were appropriate at the time of diagnosis, there were many patients who preferred clinicians to focus on the positive aspects of their care. The studies in this review suggest that palliative care involvement is likely to be more acceptable to patients in the latter two stages of the disease journey, when patients have adjusted to the diagnosis and are thinking about navigating their disease journey in the future (Figure 4). We agree with other studies which promote an individualised approach to the introduction of palliative care discussions based on ‘triggers’ or ‘turning points’ in the disease trajectory.^37,67^ Factors which should prompt clinicians to consider palliative care referral are changes in the patients’ health status, such as chest infections, onset of new symptoms, hospital admission, decline in physical functioning and the initiation of oxygen. These factors align with how patients recognised progression of their disease and therefore are points in the illness journey where patients may be more prepared to discuss palliative care and future care planning. This is especially true regarding the initiation of oxygen therapy which was viewed with apprehension and seen as a negative milestone by people with interstitial lung disease and has previously been identified as an appropriate time to involve palliative care services.^10^

Changes in health status will not always coincide with planned outpatient clinic review. Therefore, it is important that patients can contact their clinical team to discuss perceived changes in their health status to facilitate timely discussion about investigations, treatment options and potential palliative care referral. A novel rapid clinic review pathway was developed in the UK by Newcastle-upon-Tyne Hospitals interstitial lung disease service for patients who required urgent ad-hoc review.^68^ Patients were reviewed urgently in the clinic and were referred for appropriate investigations, started on treatment for intercurrent illness and many were also reviewed by palliative care services on the same day. Feedback from this service indicated that patients and their carers felt supported as they were able to be reviewed by the interstitial lung disease specialists quickly, which avoided delays which may have been fostered by repeated visits in primary care.

A prevalent theme in our review was the role of informal carers and the strain and burden of this responsibility. The physical and psychological strain on informal carers of family members with chronic disease is widely reported.^69–71^ Our study identified that carers perceived a breakdown in co-ordination of care and uncertainty about who to contact in the community. This is supported by Eriksson et al.^70^ who identified that many carers were dissatisfied with the level of formal support available and relied on their informal networks. Our study identified that patients and carers had differing information needs which changed throughout the patient journey as the disease progressed. Carers were often more direct in their need for practical information about how the disease would progress, what to expect towards the end-of-life and who to contact in the community. Sampson et al.^37^ reported that informal carers felt their role was ‘ambiguous’, resulting in them not obtaining the information or support they required. Clinicians should be sensitive to the strain on informal carers which increases alongside the patients’ clinical deterioration and seek to acknowledge and include carers in outpatient clinics and address the changing needs in their relationship.^53^ Carers may require additional information or time to ask questions separately to their relatives and this should be facilitated in an outpatient clinic.

## Conclusion

Our study identified three interconnecting stages in the interstitial lung disease patient’s journey: information seeking, grief and acceptance, fear of the future. We suggest that palliative care involvement is most appropriate in the latter two stages and should be prompted by changes in patients’ health status.

It is the role of healthcare professionals to support people with interstitial lung disease and their carers through the diagnostic process by delivering timely and accurate information whilst addressing their individual concerns. People with interstitial lung disease appreciate honest and open conversations and trust their clinical team to decide the appropriate amount of information to provide. Healthcare professionals must recognise changes in patients’ health status and initiate conversations which address palliative care needs and potential onwards palliative care referral.

Our study recognises the significant strain placed on the informal carers for people with interstitial lung disease. Future research should review how to support people with interstitial lung disease and their carers in the community.

Future research should consider timely recognition of changes in patients’ health status and how to respond to these changes in a community setting.

## Supplemental Material

sj-pdf-1-pmj-10.1177_02692163221101753 – Supplemental material for When should palliative care be introduced for people with progressive fibrotic interstitial lung disease? A meta-ethnography of the experiences of people with end-stage interstitial lung disease and their family carers Supplemental material, sj-pdf-1-pmj-10.1177_02692163221101753 for When should palliative care be introduced for people with progressive fibrotic interstitial lung disease? A meta-ethnography of the experiences of people with end-stage interstitial lung disease and their family carers by Evelyn Palmer, Emily Kavanagh, Shelina Visram, Anne-Marie Bourke, Ian Forrest and Catherine Exley in Palliative Medicine

sj-pdf-2-pmj-10.1177_02692163221101753 – Supplemental material for When should palliative care be introduced for people with progressive fibrotic interstitial lung disease? A meta-ethnography of the experiences of people with end-stage interstitial lung disease and their family carers Supplemental material, sj-pdf-2-pmj-10.1177_02692163221101753 for When should palliative care be introduced for people with progressive fibrotic interstitial lung disease? A meta-ethnography of the experiences of people with end-stage interstitial lung disease and their family carers by Evelyn Palmer, Emily Kavanagh, Shelina Visram, Anne-Marie Bourke, Ian Forrest and Catherine Exley in Palliative Medicine
