# Supplementary material for: When should palliative care be introduced for people with progressive fibrotic interstitial lung disease? A meta-ethnography of the experiences of people with end-stage interstitial lung disease and their family carers
Source: Palliat Med. 2022 Jun 11;36(8):1171–85. doi: 10.1177/02692163221101753 (PMC9446428; doi:10.1177/02692163221101753)
Supplement: sj-pdf-1-pmj-10.1177_02692163221101753 – Supplemental material for When should palliative care be introduced for people with progressive fibrotic interstitial lung disease? A meta-ethnography of the experiences of people with end-stage interstitial lung disease and their family carers [file sj-pdf-1-pmj-10.1177_02692163221101753.pdf]

Supplementary material: Search strategy

EMBASE search strategy (20/04/2022)

Ovid®

My Account | My PayPerView | Support & Training | Help | Feedback | Logged in as Eve Palmer | Logoff

Wolters Kluwer

Search | Journals | Books | Multimedia | My Workspace | What's New

▼ Search History (26)

View Saved

# ▲	Searches	Results	Type	Actions	Annotations
1	exp Lung Diseases, Interstitial/	80774	Advanced	Display Results | More ▾	
2	interstitial lung disease*.tw.	22816	Advanced	Display Results | More ▾	
3	exp lung fibrosis/	65975	Advanced	Display Results | More ▾	
4	pulmonary fibrosis.mp.	31772	Advanced	Display Results | More ▾	
5	exp allergic pneumonitis/	9156	Advanced	Display Results | More ▾	
6	hypersensitivity pneumonitis.mp.	3730	Advanced	Display Results | More ▾	
7	exp fibrosing alveolitis/	27725	Advanced	Display Results | More ▾	
8	1 or 2 or 3 or 4 or 5 or 6 or 7	119145	Advanced	Display Results | More ▾	
9	end-stage.mp. [mp=title, abstract, heading word, drug trade name, original title, device manufacturer, drug manufacturer, device trade name, keyword heading word, floating subheading word, candidate term word]	137105	Advanced	Display Results | More ▾	
10	terminal* ill*.mp. [mp=title, abstract, heading word, drug trade name, original title, device manufacturer, drug manufacturer, device trade name, keyword heading word, floating subheading word, candidate term word]	12053	Advanced	Display Results | More ▾	
11	(end adj6 life).mp. [mp=title, abstract, heading word, drug trade name, original title, device manufacturer, drug manufacturer, device trade name, keyword heading word, floating subheading word, candidate term word]	47132	Advanced	Display Results | More ▾	
12	terminal stage*.mp. [mp=title, abstract, heading word, drug trade name, original title, device manufacturer, drug manufacturer, device trade name, keyword heading word, floating subheading word, candidate term word]	3118	Advanced	Display Results | More ▾	
13	((advanced or end stage or terminal*) adj4 (disease* or ill*)).mp. [mp=title, abstract, heading word, drug trade name, original title, device manufacturer, drug manufacturer, device trade name, keyword heading word, floating subheading word, candidate term word]	207993	Advanced	Display Results | More ▾	
14	exp palliative therapy/	119886	Advanced	Display Results | More ▾	
15	palliative care.mp.	57490	Advanced	Display Results | More ▾	
16	place of death.mp. [mp=title, abstract, heading word, drug trade name, original title, device manufacturer, drug manufacturer, device trade name, keyword heading	2176	Advanced	Display Results | More ▾	
17	end of life care.mp. [mp=title, abstract, heading word, drug trade name, original title, device manufacturer, drug manufacturer, device trade name, keyword heading word, floating subheading word, candidate term word]	18260	Advanced	Display Results | More ▾	
18	terminal care.mp. [mp=title, abstract, heading word, drug trade name, original title, device manufacturer, drug manufacturer, device trade name, keyword heading word, floating subheading word, candidate term word]	33256	Advanced	Display Results | More ▾	
19	dying.mp.	44634	Advanced	Display Results | More ▾	
20	9 or 10 or 11 or 12 or 13 or 14 or 15 or 16 or 17 or 18 or 19	409461	Advanced	Display Results | More ▾	
21	8 and 20	4581	Advanced	Display Results | More ▾	
22	interview.tw.	181041	Advanced	Display Results | More ▾	
23	exp health care organisation/	1730141	Advanced	Display Results | More ▾	
24	experiences.tw.	252604	Advanced	Display Results | More ▾	
25	22 or 23 or 24	2083251	Advanced	Display Results | More ▾	
26	21 and 25	367	Advanced	Display Results | More ▾	

Contract

Save | Remove | Combine with: AND | OR

Supplementary material: Search strategy

MEDLINE search strategy (20/04/2022)

Ovid® | Wolters Kluwer

My Account My PayPerView Support & Training Help Feedback Logged in as Eve Palmer Logoff

Search Journals Books Multimedia My Workspace What's New

▼ Search History (26) View Saved

# ▲	Searches	Results	Type	Actions	Annotations
1	end-stage.mp. [mp=title, abstract, original title, name of substance word, subject heading word, floating sub-heading word, keyword heading word, organism supplementary concept word, protocol supplementary concept word, rare disease supplementary concept word, unique identifier, synonyms]	62515	Advanced	Display Results More	
2	terminal* ill*.mp. [mp=title, abstract, original title, name of substance word, subject heading word, floating sub-heading word, keyword heading word, organism supplementary concept word, protocol supplementary concept word, rare disease supplementary concept word, unique identifier, synonyms]	9148	Advanced	Display Results More	
3	(end adj6 life).mp. [mp=title, abstract, original title, name of substance word, subject heading word, floating sub-heading word, keyword heading word, organism supplementary concept word, protocol supplementary concept word, rare disease supplementary concept word, unique identifier, synonyms]	27692	Advanced	Display Results More	
4	terminal stage*.mp. [mp=title, abstract, original title, name of substance word, subject heading word, floating sub-heading word, keyword heading word, organism supplementary concept word, protocol supplementary concept word, rare disease supplementary concept word, unique identifier, synonyms]	1860	Advanced	Display Results More	
5	((advanced or end stage or terminal*) adj4 (disease* or ill*)).mp. [mp=title, abstract, original title, name of substance word, subject heading word, floating sub-heading word, keyword heading word, organism supplementary concept word, protocol supplementary concept word, rare disease supplementary concept word, unique identifier, synonyms]	97446	Advanced	Display Results More	
6	exp palliative therapy/	47622	Advanced	Display Results More	
7	palliative care.mp.	55930	Advanced	Display Results More	
8	place of death.mp. [mp=title, abstract, original title, name of substance word, subject heading word, floating sub-heading word, keyword heading word, organism supplementary concept word, protocol supplementary concept word, rare disease supplementary concept word, unique identifier, synonyms]	1228	Advanced	Display Results More	
9	end of life care.mp. [mp=title, abstract, original title, name of substance word, subject heading word, floating sub-heading word, keyword heading word, organism supplementary concept word, protocol supplementary concept word, rare disease supplementary concept word, unique identifier, synonyms]	10950	Advanced	Display Results More	
10	terminal care.mp. [mp=title, abstract, original title, name of substance word, subject heading word, floating sub-heading word, keyword heading word, organism supplementary concept word, protocol supplementary concept word, rare disease supplementary concept word, unique identifier, synonyms]	23338	Advanced	Display Results More	
11	dying.mp.	26093	Advanced	Display Results More	
12	1 or 2 or 3 or 4 or 5 or 6 or 7 or 8 or 9 or 10 or 11	199875	Advanced	Display Results More	
13	exp Lung Diseases, Interstitial/	45160	Advanced	Display Results More	
14	interstitial lung disease$.mp. [mp=title, abstract, original title, name of substance word, subject heading word, floating sub-heading word, keyword heading word,	10295	Advanced	Display Results More	
15	interstitial pneumonia$.mp.	5988	Advanced	Display Results More	
16	exp Pulmonary Fibrosis/	16046	Advanced	Display Results More	
17	idiopathic pulmonary fibrosis.mp. [mp=title, abstract, original title, name of substance word, subject heading word, floating sub-heading word, keyword heading word, organism supplementary concept word, protocol supplementary concept word, rare disease supplementary concept word, unique identifier, synonyms]	9134	Advanced	Display Results More	
18	allergic alveolitis.mp. [mp=title, abstract, original title, name of substance word, subject heading word, floating sub-heading word, keyword heading word, organism supplementary concept word, protocol supplementary concept word, rare disease supplementary concept word, unique identifier, synonyms]	379	Advanced	Display Results More	
19	13 or 14 or 15 or 16 or 17 or 18	50364	Advanced	Display Results More	
20	12 and 19	1105	Advanced	Display Results More	
21	interview*.ti,ab.	318092	Advanced	Display Results More	
22	interview/	19709	Advanced	Display Results More	
23	experience*.mp.	861810	Advanced	Display Results More	
24	qualitative.ti,ab.	203063	Advanced	Display Results More	
25	21 or 22 or 23 or 24	1203965	Advanced	Display Results More	
26	20 and 25	157	Advanced	Display Results More	

Contract

Save Remove Combine with: AND OR
