# Supplementary material for: When should palliative care be introduced for people with progressive fibrotic interstitial lung disease? A meta-ethnography of the experiences of people with end-stage interstitial lung disease and their family carers
Source: Palliat Med. 2022 Jun 11;36(8):1171–85. doi: 10.1177/02692163221101753 (PMC9446428; doi:10.1177/02692163221101753)
Supplement: sj-pdf-2-pmj-10.1177_02692163221101753 – Supplemental material for When should palliative care be introduced for people with progressive fibrotic interstitial lung disease? A meta-ethnography of the experiences of people with end-stage interstitial lung disease and their family carers [file sj-pdf-2-pmj-10.1177_02692163221101753.pdf]

Supplementary material

Illustrative quotes to support the development of second order constructs and key concepts

Key concepts<br><br>(Generated from reciprocal synthesis)	Sub-concepts	Second order constructs<br><br>(Themes developed by primary authors)	First order constructs<br><br>(Participant quotes/primary data from the studies)
**Information seeking**	**Diagnostic delay and misdiagnosis**	Diagnosis (Belkin et al)<br><br>Struggling to get a diagnosis (Duck et al)<br><br>Frustration with the diagnostic process and education received (Lindell et al)	…some kind of fibrosis….so I just kept going to the clinic and they did lung function…which…at the time….were quite low…then he decided to refer me to Dr. X's clinic and then we found out I was too bad for the trial (patient study 4)<br><br>I was diagnosed 4 years ago, but I was being treated for asthma and being overweight (patient study 4)<br><br>...my primary care physician kept hearing crackling in my lungs. So we took an x-ray that first year at my annual physical. A year later, another physical, he says 'Oh I can still hear that crackling. I don't like that. Let's get an x-ray.' So we took another x-ray. Initially he thought that perhaps I had some kind of scarring from pneumonia, but the second year, second annual physical, we went to a pulmonologist - and he requested that I do the biopsy... (patient study 9)<br><br>When my wife first found out it, she kept coughing and I kept telling her to go to the MD. She had a biopsy and the report said IPF. I "googled" it and what I read wasn't what I wanted to read. We asked if we could go to the specialty centre and were told that she wasn't sick enough. She died the next month. (husband of patient study 9)<br><br>It started about 5 years ago, but I didn't realise then that something was terribly wrong. It just came gradually. I was diagnosed with something "fibrotic" and was told that I probably had untreated asthma. (patient study 10)<br><br>It wasn't even told to us that my husband could have pulmonary fibrosis until the 12th of February. And then he was gone the 11th August. So it was very quick. (carer, study 11)

	**Information needs**	Making sense of the inexplicable (Bajwah, Koffman et al)<br><br>End of life information needs (Bajwah, Koffman et al)<br><br>Sources of information (Bawah, Koffman et al)<br><br>Struggling to get a diagnosis (Duck et al)<br><br>Tell me about the future (Holland et al)<br><br>Seeking information from multiple sources (Holland et al)<br><br>Information and disclosure (Overgaard)<br><br>Understanding of symptoms and medical interventions (Sampson et al)	I think that the information here at this place [specialty centre] is handed out differently than the information at other places. (carer, study 9)<br><br>I think the best thing would be if the hospital were a bit more aware of gathering the family and having a good talk about this. (carer, study 10)<br><br>Well I haven't got a very long future so ah, I suppose I'd like a bit of honesty from them [clinicians]....I've been sort of kept in the dark a little. (patient with IPF, study 6)<br><br>I don't understand it near as well, as I feel I need to. I was never sick my whole life, and up to just about two years ago. I started having problems and - short of breath, didn't understand why, and not until my personal physician took and x-ray of my chest and he says, you need to go to a specialist....I don't really totally understand what I have. I know what they tell me I get, but and they've explained a lot, but ...I still don't understand. (patient, study 9)<br><br>I've been told enough in the [IPF] clinic so you know… I've got copies from all the letters from the clinic as well, you know. I insisted I have a copy of them … I want to see what they are telling the GP, you know to make sure and to see what… to try and understand the diagnosis. (patient with IPF, study 12)<br><br>I kind of get a feeling that even coming to the support group is part of palliative care because we're learning every aspect of the body and how to live better and they talk about how to get through this. (patient, study 9)<br><br>I haven't sort um I haven't really discussed...um...how it will develop with anybody, but you know that may be me own fault um if I don't talk about it, to somebody, and you don't know then I've got nothing to worry about (laughs) (patient, study 2)<br><br>I think at diagnosis, obviously, you need to be told that this is not curable and it's progressive and it will end your life […] [want to have ACP conversations with] An IPF specialist, maybe a nurse practitioner in IPF. Or a respiratory therapist who focuses only on

			IPF…someone who's really knowledgeable about it. (patient with IPF, study 7)<br><br>Empowering the patient with information about what their disease is (...) eh ... what's likely to happen and the treatment so you're involving them very early on so they have a good understanding of the whole... (...)package of what's wrong with them. I think that is the single most important thing we can do (...) eh to help them understand. (ILD consultant, study 2)<br><br>I think sometimes the delivery is wrong (...) which could be a problem. Um what you don't want to do is completely say 'you're going to die' what you want to do is give them some hope... (ILD nurse specialist, study 2)<br><br>It [the internet] was helpful but I already know the information because the doctors at the hospital already told me. (patient with ILD, study 6)<br><br>I want to know what I should do to prevent it from getting worse and what is the good exercise for this situation. (patient with IPF, study 6)<br><br>…it's hard to anticipate what help I need if I don't know what's coming… I never know how doctors feel about a spouse sitting there and suddenly taking over the conversation so I generally don't ask questions. I answer questions if I'm asked…but I think that I'm there just as a support system to [partner], rather than having my own questions answered. (carer, study 12)<br><br>I actually didn't find [information on the internet] reliable. (patient with IPF, study 7)<br><br>It was so hard to find information and any doctors or nurses we did talk to had never had a patient with IPF before […] they were comparing it to COPD or other diseases that it really can't be compared to. There was no information and it felt like we were completely on our own…From what I found online, it was scary; but I figured if that was the case, then her doctor would tell us. (carer, study 7)

Supplementary material

**Grief and adjustment**	**Overwhelming symptom burden**	The extent of physical and psychological needs (Bajwah, Higginson et al)<br><br>Living with IPF (Duck et al)<br>Overwhelming symptom burden (Lindell et al)<br><br>Having a terminal disease (Pooler et al)	I'm breathless, always breathless (female patients in her 50s, study 1)<br><br>The coughing fits are so draining on him, he'll be in the middle of something, start coughing and have to sit down and catch his breath. (carer study 9)<br><br>She'll panic because although she tries not to but em she would panic because it's not nice not being able to breathe you know (husband of patient, study 1)<br><br>the symptom of breathlessness is the most significant symptom that I've come across (...) ... it could mean that they are anxious as well about the breathlessness and about whether this is what it is going to feel like when they die (ILD physiotherapist, study 1)<br><br>when it's really bad, I'd make a trade with the devil (...) because I'm so (...) flat and exhausted and [I] think well I'd rather not go on. (male patient in his 60s, study 1)<br><br>Pulmonary fibrosis, you know, is a sad and severe illness. It's a person that can't breathe, it's terrible to watch. (carer, study 5)<br><br>...when I go upstairs and sometimes I walk up, no trouble, ... cough very, very little.... others times I walk up, start coughing and then I've really got to go to the toilet and if I don't go to the toilet, well...sometimes I wet myself. (patient, study 4)<br><br>I just couldn't come to terms with it...I was getting panic attacks...two or three times a week...and used to break down...cry like a baby...there were times I just wanted to smash my head against a wall. (patient, study 4)
	**"Loss" of previous life**	Impact of disease on social activities (Bajwah, Higginson et al)	I get out of breath; I can't do the things I used to. She has taken over mowing the lawn. (patient, study 10)<br><br>I can't go anywhere […] … I don't don't [really] have a life I'm sitting indoors everyday…I used to be meet friends and have coffee and it [would] give you a bit of life back ... (female patient in her 70s, study 1)

		Reliance on others and change in relationships (Bajwah, Higginson et al)<br><br>Life with oxygen (Belkin et al)<br><br>Loss of the life I previously had (Duck et al)<br><br>Gradual tacit role shift (Overgaard et al)	I've always been active, garden, DIY, you know I'd do anything around the house. I mean there was a little job there yesterday and I had to get Christine to (do it)...I sort of direct operations now, but physically do it, No. (patient, study 4)<br><br>So it's getting around I think is the big change....actually doing jobs.....you've got to think before you do anything, if you want to....just nip to the shops, you've gotta think 'have I enough liquid oxygen? Have I filled the bottle? Have I done this? Have I done that?' (patient, study 4)<br><br>I was gardening after 36 years of er er working in management, and then starting to chop down trees and digging um holes in the ground in the open countryside, the sun beating down and all the plants and everything, I was loving every minute of it and now I can hardly get into my own garden, I bend down to pull a weed out , I have to take 10 minutes to get up off my knees again... (patient in his 60s, study 1)<br><br>The biggest thing for me has been the change...to lose out on being able to work...to be honest with you...the finances. I've been a worker all my life. Do I need to sell my home? It's hard to see my wife taking care of me. (patient, study 9)<br><br>Over 30 years we've been playing cards, alternate weeks....playing poker and that's stopped 'cause she can't concentrate when we're playing. (carer, study 4)<br><br>I know my husband, he is just tired of being sick...being sick, and your whole life revolving around it...they just want to be normal. (carer, study 3)<br><br>we used to go swimming together and now we’re not going swimming together. And we used to go walking for the whole day and now we’re not... ... We’d go out all the time. (carer, study 12)
	**Carer strain**	Reliance on others and change in	Coughing! Coughing! People would ask why didn't you go to a different bedroom so you didn't have to hear the cough, but I would feel bad, I couldn't tell him. I was going to work on 2-3 hours of sleep. (carer study 9)

		relationships (Bajwah, Higginson et al)<br><br>Living with patient as disease progresses (Belkin et al)<br><br>Feeling strain and responsibility (Pooler et al)<br><br>Coping strategies and carer roles (Sampson et al)	In the beginning I constantly watched him...and the first time he fainted, I must admit, I was really scared. (carer study 9)<br><br>He can't do a thing, we don't talk about it. I do what has to be done. (carer study 10)<br><br>trying to keep him thinking positively and just trying to get through each day and some days it's not too bad and other days it's a real struggle...he's becoming more and more depressed... (wife of patient, study 1)<br><br>... where I find it difficult is I want to do everything for him. You tend to want to say no you stay there you sit there, don't move, I'll do it, and then that makes him cross as well and he keeps saying 'I'm not dead yet'... (wife of patient, study 1)<br><br>I have to cook and everything. There's no such thing as a vacation. (carer, study 10)<br><br>he can't cope with it, and I'm there, so he'll tend to vent whatever he's feeling at me, um verbally or [...] you know [...] I just won't talk um so it is a strain, so it puts a strain on the whole relationship really (sigh) ... sometimes he gets quite emotional about it all, and other times her gets quite nasty. (wife of patient, study 1)<br><br>I found [dying at home] difficult, because he was very ill and it required a lot of work. I didn't always have the stamina or knowledge of how to manage, so sometimes we barked at each other. When I look back, I should have had help....so we could have enjoyed each other. (carer, study 5)<br><br>I think we are so used to doing things for ourselves, we don't know what people can help with sometimes. [...] Even knowing who to call, or someone to call, would be really nice because when you're he caregiver it's not 9 to 5. It's 24 hours. If something happens in the middle of the night, it happens in the middle of the night. (carer, study 11)<br><br>Your life [as the informal carer] keeps getting smaller, which is really difficult. (carer, study 3)

			There isn't anything you can do. That's the frustrating part for me, when he really starts coughing and you want to do something to help...and there is nothing. (carer, study 3)
**Fear of the future**	**Oxygen as a "negative milestone"**	Life with oxygen (Belkin et al)<br><br>Living with IPF (Duck et al)<br><br>Timely identification of changes in health status and functional activity (Sampson et al)	My world crashed for a second time when oxygen was started, it was chaos. (carer study 10)<br><br>I quit work last year when I started to need oxygen. I called in sick, partly because I psychologically felt it would be wrong to go to work, and so on, with oxygen and a blue face. I don't think this would go over well in the advertising business [laugh]. (patient, study 10)<br><br>well, and that's just like using oxygen, getting into a wheelchair, you know, the whole pride. it impacts that person's identity and their version of what strength is. And so like resistance to using oxygen or taking it out in public....oh what a fight that was. (carer, study 9)<br><br>...with the oxygen on at least I can get around, not as fast as normally, but I can get around...and I don't seem to cough as much. (patient, study 4)<br><br>...I couldn't sit here this long and talk to you without oxygen. (patient, study 4)<br><br>[Having to deal with oxygen] destroys everything, I think, in your normal life that you [once] did. (carer, study 3)<br><br>We are not dealing with oxygen yet, and I hope we can put that off a long time, but in reality, we know it probably will happen at some point....it is terrifying to me and to him. (carer, study 3)<br><br>[Patient] can't walk, so I didn't know if maybe the oxygen would help him. I would like to know that. And I think [patient] thinks, once he's gone on that… I think he thinks that's the beginning of the end. That it's a new stage. That it's not the beginning of being able to do more. I would look at it as a beginning to be able to do more and I think he looks at it as the beginning of the end. That you've given in to something maybe or… Because it's not… if it's going to help you, it's not giving in, is it? (carer, study 12)

			I think he feels it’s a failure or that he’s, perhaps that he’s getting worse if he uses it. But I said to him, well you know, that’s what they said, when you move around you need the oxygen. (carer, study 12)<br><br>Ya, so when I get fatigued , if I hook up to it after five minutes I kind of clear myself up. So obviously it is lack of oxygen that is causing all of that fatigue. (patient, study 8)<br><br>The fact that you can't go anywhere for too long without the oxygen (patient, study 8)<br><br>When he was getting short of oxygen and we needed to change the amount, I knew. Because he was just more irritable. (carer, study 8)<br><br>It's as their oxygen levels climb. They start to realise that they’re getting sicker. And that is often the only symptom. (HCP, study 7)
	**Perceptions of palliative care + advance care planning**	End of life planning, decision making and care (Bajwah, Koffman et al)<br><br>Co-ordination of care (Bajwah, Koffman et al)<br><br>Tell me about the future (Holland et al)<br><br>Perceptions (Insufficient information, conversations occur	I believe palliative care should be with you from when you're diagnosed, saying "You know what, you're terminal but we are going to help you live until you die". I figure you should enjoy every minute of your life. We don't know, you could live two years, three years, you could live six months or a month. But if you could access somebody and say, "This is the disease I have. What steps can I do to stay in my home, be happy in my home and as my disease progresses, who can I have, whether it be nursing staff, any access to anything that's going to make my life at home easier for myself and my caregivers." (patient, study 11)<br><br>... I didn't know this [focus group] had anything to do with palliative care, and I do have a problem focusing on the fatality. [rolled seat back away from group] in answer to your question, YES I would rather focus on the positive. You hear terminal once; you don't need to hear it again. So...if there are positives, you focus on that. (carer, study 9)<br><br>I'm afraid if we talk about the future, he will think that I know he is going to die. (carer, study 9)

		late) (Kalluri, Orienstein et al)<br><br>Recommendations (Have earlier conversations, provide information, have open conversations, plan for end of life care) (Kalluri, Orienstein et al)<br><br>Engaged in anticipatory planning (Kalluri, Younus et al)<br><br>Reluctance to engage in ACP (Lindell et al)<br><br>Planning goals and wishes for care (Pooler et al)<br><br>Timely identification of changes in health status and functional activity (Sampson et al)	They talked to us about advance care planning. He understood how it was and me too. And he wanted to die at home so that's why I took care of him at home. I was involved in that I was accepting of his wishes. And I felt I needed to respect him. (carer, study 11)<br><br>Their honesty in their conversation was huge as well. Being able to look my mom in the eye and tell her that she's dying and that she will be dead within the year. And then, taking the time to sit there and hold her hand and wait until she was ready to talk, and answer questions, and let her know what death was going to look like, (pause) I think is also really big. (carer, study 11)<br><br>As far as the will, I don't have any kind of will made up. We've talked about it. We - things as far as what you want done towards resuscitate, no resuscitate. We kinda talked about that. (patient, study 9)<br><br>And that was the last thing I could give him, it was to let him stay at home. It was his biggest wish and he was grateful that I would help. (carer, study 5)<br><br>Well, with a hospice you're going to die. Right soon, within six months, usually. That's what I understand. And palliative care s just to help make you comfortable. But we know that you die with this disease, so what is the difference? Is it a longer time you can be on palliative care? Or people help you deal with the disease? I don't know. I'm just guessing. (carer, study 9)<br><br>I don't expect to be looked after [before dying], I just expect that sooner or later I'm going to pop off the edge of the cliff. So I won't need looking after. (patient, study 2)<br><br>I think it is extremely difficult for a person who is responsible for a group's therapy to individualize a discussion about end of life. So I don't think that's a natural context for that very important discussion. (respiratory consultant, study 6)<br><br>I'd like to know more about it. And maybe without [patient] being there, I would like to know what can happen at the end… I don't want to sit with [patient] and know that. To see that I am devastated. (carer, study 12)

			Well I knew somebody that's got this disease… and from what I can understand he's reasonable sitting in a chair, but other than that it's hopeless…he's got oxygen permanently and he's got a stair-lift to go up to bed. Now he's got a wife so he obviously has someone to take care of him but I'm widowed so don't have anyone here, so you know when you get to that stage that's the thing that I find a little concerning… (patient with IPF, study 12)<br><br>And I know what my plan is - I'm going to call the ambulance - if I can; and then my daughter will find me, and my friends and they will call my sister; and then they will look after me and decide whether they are going to move me or leave me there; and then that's the end. (patient, study 8)<br><br>Yup, we have our wills made; we have our paperwork done....We've got all end of life I think cared for. We have a plan. He's going to stay home. We are going to do everything here and we have heard that Dr J. will support us in that so that's all we need. (carer, study 8)<br><br>Being honest and telling me what's going to happen. It's very important. It's one of my biggest fears […] I do feel much better just knowing, I was afraid of the end. Of how violent it could be. (patient with IPF, study 7)<br><br>I found it a lot easier to have those conversations when we were both given the same information because if one of you is at home and one didn't really want to upset the other one by telling what you had heard; but if you're both there and both hear the same things it's easier to talk about…this disease doesn't hold back so I don't think the pamphlet should either. It might be scary to some people, but it is what they need to hear. (carer, study 7)
	**Inconsistent co-ordination of care**	Co-ordination of care (Bajwah, Koffman et al)<br><br>Stage 1: before death - the final days	I think they try to liaise between each other but it so often falls apart … there really is a short coming um getting information from one aspect of the medical profession to the other. (wife of patient study 2)<br><br>The breakdown of communication over in [local hospital] has been (…) dreadful for someone who's got (…) supposedly um … you know a terminal illness, it's been dreadful (daughter of patient study 2)

		(Emotional ambivalence, physical surroundings) (Egerod et al)<br><br>Comfort in speciality centre because of resources (Lindell et al)	We need to really (…) review the way we think about people who've got (…) um rapidly progressive non malignant disease or people who are literally dying from non-malignant disease, the kind of communication we expect around cancer (…) really should happen around (…) these other diseases, so take IPF (…) I think the quality of communication has got to be a lot better, these people don't get treated properly (GP study 2)<br><br>And then they promised that he would go to his usual hospital, but suddenly one morning the nurses said that he wouldn't go there. I actually think he got angry and then he couldn't breathe. (carer, study 5)<br><br>They [hospice] were not honest when they said they had oxygen. We were surprised that we had to bring our own equipment. (carer, study 5)<br><br>I think she wasn't prepared. She didn't see herself as palliative. The respirologist involved kept saying "just send her to acute". The client has expressed a desire to die at home. But the respirologist involved wasn't treating her like she was palliative, so didn't have in place any kind of crisis dyspnoea management or orders for how she would be managed at home while actively dying. (HCP, study 7)
	**Dying**	End of life (Belkin et al)<br><br>Stage 2: dying – the final hours (Timing, location and process of death) (Egerod et al)<br><br>Nearing the end (Pooler et al)	Um I mean I have never seen quite so much phlegm (laughs nervously) and he was literally choking on it, he was deeply blue and there was a sense of sort of hopelessness that nobody could actually do anything about it and I thought there probably were few few worse ways to die than that when I saw it in that instant, sort of haunts me a bit today. (Palliative care consultant, study 1)<br><br>But I would have liked to know how bad it was. My daughter and I left at six and were called up very late. It was a shock. (carer, study 5)<br><br>Afterwards I was surprised and sorry he died so fast. If I had known I wouldn't have gone home to sleep. If they had known at the hospital, I would have liked to know. (carer, study 5)

			I knew he wasn't afraid of dying. He said so, but he was afraid of the process, and worried that he would suffocate. (carer, study 5)<br><br>The nurse came to our home several times during the day and I slowly understood that this was it...I called the closest family and we stayed with him all day, supported by the nurse...He never woke up. We held his hand, talked to him, told him we loved him and that now he could say goodbye. We all felt that he had suffered enough and that he needed peace. My greatest worry was that I couldn't keep my promise to him that he wouldn't suffocate...he took his last breath at three in the morning, death came peacefully and it comforted us....I turned off the oxygen, his life line, and feared he would gasp for breath and panic. His usual nurse helped us the next day...It was a good ending. (carer, study 5)<br><br>Even though I tell people my husband died at home, that is not for everybody and trust me I was very resistant for a very long time. But we don’t have many options, it's either hospital and intubated and we don't have a major hospice centre. So you're very limited in what you can do. And I couldn't imagine having to sit there and have him be intubated when he was so happy right until the very last minute of his life, by being at home. But to make that choice, it's a very hard choice to make, very hard. (carer, study 11)<br><br>Do I just go to hospital, and I just gasp until I die? But being reassured by them that I can have a home death, there can be drugs that can help me calm down, and people there…And how caring and supportive the doctors can be. […] So it’s not scary at the end. (patient with IPF, study 7)<br><br>I think there is a lot of fear with the changes that the body goes through when someone is like actively dying; the noises people make and the way people look. That would prepare people – like what is normal and what is alarming. It would give them some knowledge to face those things with less fear. (HCP, study 7)
